# An fMRI-informed EEG model of the amygdala is associated with salience network dynamics during naturalistic emotional stimulation

**DOI:** 10.1038/s41380-025-03418-x

**Published:** 2025-12-15

**Authors:** Talia Brandman, Yaki Stern, Guy Gurevitch, Tal Harmelech, Gal Shoval, Oded Kraft, Aron Tendler, Talma Hendler

**Affiliations:** 1 GrayMatters Health, Haifa, Israel; 2 Sagol Brain Institute, Tel Aviv Sourasky Medical Center, Tel Aviv, Israel; 3 Gray Faculty of Medical & Health Sciences, Tel Aviv University, Tel Aviv, Israel; 4 Child and Adolescent Division, Geha Mental Health Center, Petah Tikva, Israel; 5 School of Psychological Sciences, Faculty of Social Sciences, Tel Aviv University, Tel Aviv, Israel; 6 Sagol School of Neuroscience, Tel Aviv University, Tel Aviv, Israel

**Keywords:** Biomarkers, Neuroscience

## Abstract

The amygdala-derived EEG-fMRI pattern (Amyg-EFP) is an EEG model of fMRI amygdala activity with demonstrated therapeutic implications in neurofeedback interventions aimed at emotion regulation. Yet, its distributed neural correlates and potential relevance for emotional processing, independent of active modulation by neurofeedback, remain unexplored. This study aimed to characterize the distributed brain activations captured by the Amyg-EFP during passive processing of naturalistic emotional stimuli. Using simultaneous EEG-fMRI during stimulation with emotional movie and music excerpts in 71 healthy participants, we examined the spatiotemporal coupling between the Amyg-EFP and fMRI activations across the brain. We further measured the correlation of the Amyg-EFP with individual emotional reactivity ratings of the movie stimuli. Results revealed a correspondence between Amyg-EFP signal modulation and salience-network dynamics, tracking the anterior insula, dorsal anterior cingulate cortex, and salience-coactivated amygdala subregions. This association was specific to the Amyg-EFP, as a control EFP derived from the ventral striatum showed a different coupling pattern. In addition, the Amyg-EFP, but not the control EFP, was associated with emotional reactivity ratings of movie stimuli. Together, these findings tie localized amygdala activity captured by the Amyg-EFP, with broader salience-network dynamics and with individual reactivity, during the processing of emotional stimuli. Thereby, this study highlights the relevance of the Amyg-EFP for emotional reactivity, and its potential as a target in brain-based interventions for psychiatric disorders characterized by emotional dysregulation.

## Introduction

Psychiatric disorders have traditionally been diagnosed based on clinical observations of symptoms and signs, an approach limited in its ability to capture underlying neurobiological mechanisms [1]. The Research Domain Criteria (RDoC) initiative proposes a shift towards characterizing psychopathology along dimensional constructs grounded in neural circuits and processes [2–4]. This approach aims to uncover transdiagnostic neural markers that could inform interventions that target mental processes, driving the pursuit of neuroanatomically precise and functionally valid biomarkers [5–9]. Emotion regulation, a crucial process for maintaining mental health, represents one such dimensional construct [10]. Emotion regulation refers to the ability to modulate the intensity, duration, and expression of emotional responses in a context-appropriate manner. Disturbances in emotion regulation are transdiagnostic, underlying various psychiatric disorders, including depression, anxiety, and post-traumatic stress disorder (PTSD) [11, 12]. Previous evidence implies a distributed network of brain regions that may be involved in processes of emotion regulation, including the amygdala, insula, ventromedial and ventrolateral prefrontal cortices [13, 14]. Furthermore, following treatment, improved emotion regulation skills are not only reflected in behavioral and clinical measures, but can also be detected through neural measures, such as amygdala activity and amygdala-prefrontal connectivity [13–15]. For example, improved emotion regulation was associated with larger differences in amygdala response to negative versus neutral images [13], and with larger inverse amygdala-prefrontal connectivity during cognitive appraisal [14].

One way to therapeutically target brain processes related to emotion regulation, including the amygdala, is Neurofeedback (NF), a self-neuromodulation procedure in which individuals learn how to regulate their own brain activity in a certain direction [16, 17]. NF training is based on continuous measurement of brain activity from an assigned target, leading to contingent quantitative feedback about its modulation relative to a baseline, given to the trainee through an interactive feedback interface [18]. The efficacy of NF for treating a specific mental dysfunction depends on its accordance with a relevant neural target associated with an underlying mental process [9]. Given the established association of the amygdala with emotional reactivity and regulation [12, 19, 20], it could be considered a highly relevant structure for NF targeting of these processes. Studies using functional magnetic resonance imaging (fMRI) have demonstrated the feasibility and utility of amygdala-targeted NF, by showing that individuals can learn to self-regulate their amygdala activity to alleviate clinical symptoms of PTSD, anxiety or depression [17, 18]. However, for NF to be widely used, it must satisfy not only the requirement for neuroanatomical precision and process-relevance of the neural target, but also provide measurement scalability, which poses a challenge for fMRI-based NF. A scalable alternative is EEG, but it is inferior to fMRI in its neuroanatomical precision due to its lower spatial resolution and more so for activation of deeply located nuclei such as the amygdala.

Recent advances in multimodal neuroimaging and computational methodologies have enabled the development of precise neural-signal models that capture core neurocognitive processes underlying psychopathology, intended to guide translational psychiatry [21]. Along these lines, the EEG-fMRI Pattern (EFP; also known as the Electrical FingerPrint) approach uses computational modeling to combine the precision and scalability of two separate modalities, in order to offer an effective solution for NF therapy [22–24]. Specifically, the EFP is an EEG model trained on simultaneously acquired EEG and fMRI data to predict the fMRI activation of a specific brain region or network, which can then be applied to new individuals without the need for MRI scanning. By analytically linking scalp-recorded EEG signals to simultaneously acquired localized fMRI activity, the EFP method utilizes the scalability of EEG, while achieving higher neuroanatomical precision and functional validity relative to traditional EEG sampling methods used in NF [24, 25]. Utilizing this approach to target the amygdala, an EEG model was trained to predict amygdala fMRI activation, resulting in the amygdala-derived EEG-fMRI pattern (Amyg-EFP) [23, 25], shown to correlate with amygdala blood-oxygen-level-dependent (BOLD) activity in new datasets [22, 26].

In several clinical trials, repeated sessions of Amyg-EFP NF have been found to alleviate symptoms in disorders characterized by emotional dysregulation, particularly PTSD [27, 28], as well as Fibromyalgia [29], Premenstrual Dysphoric Disorder [30], and adult ADHD [31]. In addition, previous work from our group demonstrated that NF training down-regulation of the Amyg-EFP improved neural and behavioral measures affected by emotion regulation, more so than traditional whole-brain EEG NF methods [24, 32]. Specifically, in post-training fMRI, repeated sessions of Amyg-EFP NF had improved capacity for self-neuromodulation of the amygdala [24] and reduced amygdala reactivity to negative emotional stimuli [28]. Behaviorally, the training improved emotion regulation indices in an emotional stroop task [24], and in self-reports of alexithymia [22]. These findings support the usefulness of the Amyg-EFP as a target for NF interventions aimed at emotion regulation. Yet, our understanding of the Amyg-EFP signal itself is limited by the scarcity of studies examining its distributed neural correlates independent of active NF.

To date, it has been realized that successful down modulation of the Amygdala during fMRI-NF corresponds to a distributed network of activation beyond the target [32]. However, it is unclear whether such additional network recruitment is part of the NF learning process (e.g. a general regulation-task network), or related to the actual recruitment of the target. We therefore asked whether, during naturalistic emotional stimulation, the Amyg-EFP signal corresponds with emotional reactivity and with brain activation related to emotional reactivity within or beyond the amygdala. To address these questions, in the current study we examined the distributed fMRI representation of simultaneously acquired Amyg-EFP signal during audiovisual emotional stimulation, as well as the correspondence between the Amyg-EFP and participants’ emotional ratings of the same stimuli. By characterizing the distributed neural correlates of the Amyg-EFP, this study aimed to enhance our understanding of the neurobiological basis of this fMRI-guided EEG model of amygdala activity and its relevance for neurocognitive processes of emotional reactivity.

We used simultaneous EEG-fMRI and data driven analytics to examine the spatiotemporal coupling between the Amyg-EFP signal and whole-brain fMRI BOLD activations during emotional stimulation with video clips, movie scenes and music excerpts. These naturalistic stimuli were aimed to induce emotional processing in a manner that fluctuates as in real-life events, thus also naturally engaging processes of emotional reactivity and regulation. Though we did not directly measure emotion regulation modulation, we assessed emotional reactivity to the movie stimuli, as a subprocess of emotion regulation [20]. A behavioral emotional rating task, performed immediately following scanning, provided a psychological proxy of individual emotional reactivity while viewing the stimuli. To further examine the neuroanatomical specificity of the Amyg-EFP signal, we compared the whole brain fMRI correlates of the Amyg-EFP to those of a control EFP model derived from ventral striatum activity (termed here VS-EFP). This was tested in two independent groups of participants to assess replicability of data-driven neuroimaging findings.

## Materials and methods

### Ethics statement

Study protocol and all related procedures were approved by Tel-Aviv Sourasky Medical Center Institutional Review Board prior study initiation (7_0030-23, 02/03/2023; 7_0239-23, 01/08/2023). Per 21 CFR 812.28(b)(7), the study was performed in compliance with the requirements of the Ethical Committees (EC). Per 21 CFR 812.3(t), the EC that reviewed the study was responsible for ensuring the protection of the rights, safety, and well-being of subjects involved. The study was performed in accordance with the declaration of Helsinki and was conducted in agreement with International Conference on Harmonization (ICH) guidelines on Good Clinical Practice (GCP). All subjects provided written informed consent prior to being screened. The informed consent documents met the requirements of 21 CFR 50.20 and contained the information required by 21 CFR 50.25(a), and 21 CFR 50.25(b). The EC confirmed and approved the informed consent document.

### Participants

Thirty-six participants (19 male, age 18–60, M = 31.8, SD = 11.0) were included in the test group of the main study. The replication group included 35 participants (15 male, age 19–59, M = 30.2, SD = 8.9), as part of a separate ongoing study (no subject overlap), from which we obtained the earliest available data. The sample size of each group was chosen to exceed our previous work validating the Amyg-EFP within pre-defined brain regions [24], in order to allow enough power to correct for multiple comparisons in voxel-wise whole brain analyses. All participants were healthy individuals with no pre-existing medical conditions and with normal or corrected-to-normal vision and hearing, who had passed the standard screening requirements for MRI scanning. Main additional exclusion criteria included past neurological or psychological clinical diagnosis, as well as major brain injury, or current enrollment in a therapeutic clinical study.

### Stimuli

Three types of naturalistic stimuli were used to probe a range of emotional responses.

Emotional video clips. We selected short video clips from the alancowen.com database of emotionally-tagged videos [33]. The database includes 2185 short videos, ranging between 1–15 s long, which had been rated on 27 different kinds of emotion. Three categories of emotional clips were extracted from the database, including videos rated high on fear (Fear condition), high on joy (Joy condition), or low on all core emotions (Neutral condition).

Emotional movie scenes. Two movie excerpts were selected: (1) The farewell scene from the movie Stepmom (Columbus, 1998), portraying a terminally-ill mother discussing her future departure with her children. This scene was chosen for its strong evocative induction as previously demonstrated in limbic reactivity [34]. (2) A narrated scene of snakes chasing after an iguana, extracted from the nature documentary Planet Earth II (BBC, 2016), characterized by high intensity and suspense.

Musical piece. A 7-min piano track from the soundtrack the movie The Hours, by Phillip Glass, previously tested for limbic reactivity [35].

### Procedure

**Fig. 1:**
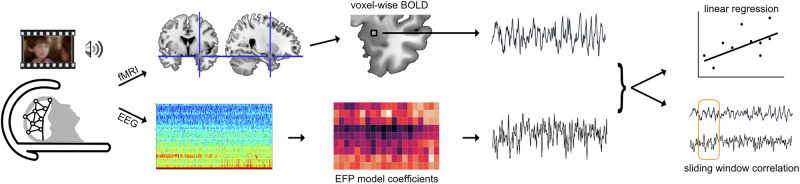
Schematic illustration of the current study design and analysis. Concurrent EEG-fMRI was acquired during naturalistic audiovisual stimulation, including emotion-tagged (fear, joy or neutral) video clips, emotional movie scenes and a classical musical piece. The fMRI signals were preprocessed to yield the voxel-wise BOLD time-course. The EEG signals were processed and weighted with existing EFP model coefficients [25] to yield the EFP time-course. To examine the relationship between BOLD and EFP we performed a GLM analysis using linear regression over the entire task time-course, as well as a sliding-window correlation analysis.

Concurrent EEG-fMRI data were acquired while participants underwent naturalistic emotional stimulation tasks, as illustrated schematically in Fig. 1. Participants arrived at Tel Aviv Sourasky Medical Center and after filling out the consent form and related questionnaires were fitted with the EEG cap, followed by a short presentation with instructions for fMRI participation and tasks. Inside the MRI scanner, participants underwent anatomical and functional scans. Three passive stimulation tasks were used for the current study: (1) viewing emotional video clips, (2) viewing emotional scenes from movies, (3) listening to classical music. Additional scan data including a reward task unrelated to the current research objectives were not analyzed here.

Emotional video clips. The video clips were presented in separate condition blocks. The Neutral clips were split into two blocks of 55 sec each, whereas Joy and Fear each consisted of one block of 110 sec. Each block presented the clips consecutively with 1-sec intervals (fixation screen) between clips. Each run began with a 5 sec fixation screen, followed by the four stimulation blocks with 15 sec fixation screen between each pair of blocks (total 380 sec). The task was run twice, with different block orders. Each clip was presented one time per run.

Movie scenes. The two audiovisual scenes were presented in a single run, beginning with 5-sec fixation screen, followed by the Stepmom scene for 501 sec, 20 sec fixation interval, Snakes scene for 321 sec, ending with 20 sec fixation screen (total 867 sec).

Musical piece. The Glass piece was presented in a single run, beginning with 5-sec fixation screen, followed by 423 sec of music and ending with 20 sec fixation screen (total 448 sec).

### fMRI acquisition

Structural and functional MRI scans were performed in a 3 T Siemens MAGNETOM Prisma scanner (Siemens, Erlangen, Germany) with a 20-channel head coil. Anatomical scans consisted of 3D T1-weighted MPRAGE sequences with a 0.9 mm iso-voxel to provide high-resolution structural images (repetition time (TR) / echo time (TE) = 1980/2.62 ms, flip angle (FA) = 8°, field of view (FOV) = 224 × 224 mm).

Functional scans were acquired using a T2*-weighted multiband accelerated EPI pulse sequence provided by Center for Magnetic Resonance Research (CMRR), with a single band reference image for better registration to the anatomical scan [36] (TR/TE = 1500/34.2 ms, FA = 74°, voxel size = 2.2 mm3, FOV = 220 × 220 mm, 57 slices per volume, multiband (MB) factor = 3). A total of 299 volumes were acquired for the music listening run, 578 volumes for the movie run and 254 volumes for each of the emotional clips runs.

Additionally, reverse phase encoding spin echo image pairs were acquired to correct susceptibility distortions in the functional images [37] (TR/TE = 8000/66 ms, FA = 90°, voxel size = 2.2 mm3, FOV = 220 × 220 mm).

### fMRI preprocessing

Data were preprocessed using fMRIPrep FMRIPrep22.0.0, [38], including slice-time correction, spatial realignment, motion correction (using the ‘mcflirt’ module of FSL), co-registration and artifact correction. Data were transformed into MNI space, spatially smoothed with a 6 mm Kernel, and corrected for motion artifacts automatically using independent component analysis (ICA-AROMA). Anatomical data were segmented, and the resulting gray-matter probabilistic map was used to determine the mask for whole-brain analyses. See Supplementary Methods for a full description of functional data preprocessing procedure given in the fMRIPrep output.

### EEG acquisition

EEG data were acquired using two battery-operated MR-compatible EEG amplifier (Brainamp MR Plus, Brain Products GmBH, Gliching, Germany) and the BrainCap electrode cap with sintered Ag/AgCl ring electrodes, providing 63 EEG channels and 1 electrocardiogram (ECG) channel positioned according to the 10–10 coordinate system. Each channel’s raw signal was amplified and sampled at 5 kHz and was online referenced to a fronto-central position (AFz). Data were recorded using the Brain Vision Recorder software (Brain Products GmbH, Gliching, Germany).

### EEG preprocessing

EEG scanner related artifacts were removed in several steps. First, an adaptive average artifact subtraction method (AAS [39],) implemented in Brain Products Analyzer Software (Version 2.2, Brain Products GmbH) was used and data were downsampled to 250 Hz. Next, data were bandpass filtered with a 4th order zero phase shift butterworth filter between 0.75–70 Hz. Cardiac R-peaks in the ECG signal were detected semi-automatically. Each r-peak was visually verified and, when necessary, manually adjusted to correct both false-positive and false-negative detections. Next, adaptive template subtraction [40] was used to remove BCG artifacts time-locked to the r-peak of the QRS complex. A 4 Hz wide notch filter centered at 33 Hz was further applied to account for a periodic scanner-related noise in that frequency. Finally, independent component analysis (ICA) was used to identify and discard eye-movement related components, according to a visual inspection of both the spatial topography and time-frequency content of each IC.

### EFP calculation

The Amyg-EFP and VS-EFP signals were calculated offline using a statistical model previously developed to enable the prediction of localized activity in the amygdala and ventral Striatum BOLD. The two models’ basic features are the Time-Frequency decomposition of the EEG in certain time windows (As in Meir-Hasson et al. [23, 25]). This was originally accomplished by applying machine learning algorithms on EEG data acquired simultaneously with fMRI and validated on an external dataset (As in [24].

For the Amyg-EFP, a 12-seconds long raw EEG segment of Pz electrode referenced to FCz electrode was notch-filtered for 50 Hz line interference, and then converted to a time-frequency representation using the Stockwell-transform. The time-frequency representation was binned to ¼ second, and the frequency domain was reduced to 10 frequencies. Each resulting time-frequency bin was then multiplied by a predefined weight and summed to a final estimate of the Amygdala BOLD signal [23, 25]. The VS-EFP was calculated by adapting the procedure used for Amyg-EFP calculation and the VS-EFP presented in a previous study [41].

### Generalized linear model analysis (GLM)

**Fig. 2:**
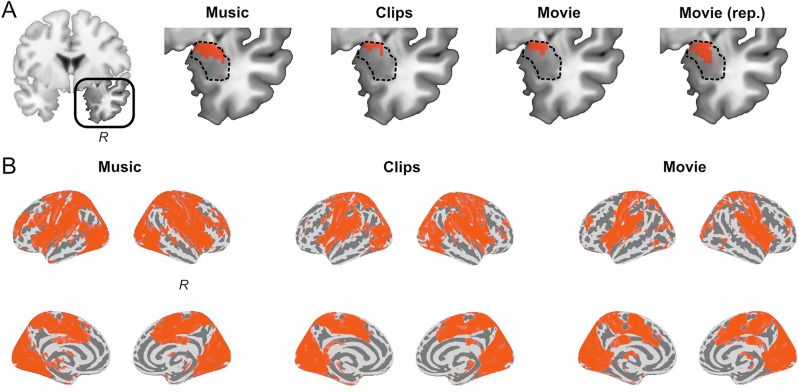
BOLD activation coupled with Amyg-EFP signal fluctuations. Results of linear regression of voxel-wise BOLD to Amyg-EFP time-course in each of the tasks, displaying above-chance voxels (P < 0.05, FDR corrected) (**A**) within the right amygdala (dashed outline) and (**B**) across the brain, displayed on inflated template brain image. Replication data denoted ‘rep’.

Whole-brain first and second-level GLM were performed using the Nilearn toolbox [42]. Signals were detrended, high-passed at 0.001 Hz and demeaned across time for each voxel, and masked using the individual probabilistic gray-matter map (in MNI space). Linear regression was performed for each subject along the full time-courses of each task, between the BOLD signal and the EFP regressor, separately for each EFP. The resulting whole-brain effect-size maps (one per task) were statistically tested at group level (single-sample t-test, 2-tails), and corrected for multiple comparisons using the false discovery rate (FDR) among all voxels, and among amygdala voxels as defined by AAL3 atlas [43] (Fig. 2A). In addition, to compare between the regression effects of the two types of EFP (Amyg-EFP and VS-EFP), the effect size maps of each participant were contrasted, and the difference statistically tested at group level (paired-samples t-test, 2-tails), with FDR correction across all voxels.

### Individual region-of-interest (ROI) fMRI selection

Within the amygdala we selected two groups of voxels based on the strength of their association with a salience network seed, during the Fear block of the first run of the Emotional Clips. For each participant, we measured the correlation between each voxel of the amygdala (as defined by the AAL3 atlas; [43]) and the mean response (across voxels) of a seed region (4-mm radius) in the right anterior insula (MNI coordinates: 36, 18, 4) intended to probe salience-network related activity [44]. This resulted in a Pearson correlation coefficient for each voxel of the amygdala, for each participant. Based on these scores, a salience-related amygdala cluster was defined as voxels whose correlation coefficients were above the 85th percentile, and at least above 0.2. A non-salience amygdala cluster was defined as voxels whose correlation coefficients were below the 15th percentile, and at least below 0.2.

### Nonparametric testing of correlation coefficients

To examine the correspondence between Amyg-EFP signal fluctuations and BOLD fluctuations in the amygdala at the individual subject level, we tested their correlation dynamics within each of the functionally-defined amygdala subregions described above. To test whether a participant’s amygdala subregion was correlated with the Amyg-EFP beyond chance, we compared the distribution (across voxels) of real correlations with a simulated null distribution, calculated as follows.

Pearson correlations were computed between the Amyg-EFP time-course and the BOLD signal of each voxel in the ROI, along a sliding time-window of 10, 20, 30, 40, or 50 repetitions (TR), in 1-TR steps (e.g. window 1: 1–10, window 2: 2–11, …). For each voxel, the resulting correlation coefficients were averaged across windows. This yielded the real-correlation distribution (separately for each window size) of size n, where n is the number of voxels in the ROI.

To obtain the null distribution, windows of the EFP time-course were shuffled, but not the repetitions within windows (e.g., window 1: 53–62, window 2: 4–13, …). Correlations were then computed using the same steps as for the real distribution. This permutation procedure was repeated 100 times to obtain a null distribution of the size 100 x n, where n is the number of voxels in the ROI.

Chance level was determined for each individual ROI by the 95th percentile of its null distribution. Thus, a single subject’s ROI-to-EFP correlation was considered above-chance if the median of the real distribution was higher than the 95th percentile of the null.

For presentation purposes, we multiplied the real distribution frequencies by 100 to match the number of null frequencies, such that they can be displayed side by side.

### Behavioral rating of emotional movie scenes

To measure natural fluctuations in emotional response, we asked participants to rate the two movie scenes presented in the movie task, while viewing them a second time outside the scanner. Of the total 71 fMRI-EEG participants, 61 completed the rating task. Participants were asked to refer to the emotional experience of their first viewing (inside the scanner), and rate it continuously throughout the timeline of the movie task on a subjective emotional reaction scale (“How does it make you feel”) from 1-very negative to 7-very positive (4- no emotional reaction). Emotional ratings were centered to 0, converted to absolute emotional reactivity (i.e., absolute distance from neutral) and averaged across the timeline of each of the movie scenes. For each participant we then calculated the difference in emotional reactivity score between the two movies (Snakes scene minus Farewell scene, see Stimuli). Pearson correlations were calculated across subjects between the magnitudes of difference in movie ratings and the magnitudes of difference in EFP response (mean amplitude across time) to the two movies.

## Results

### Amygdala and whole-brain fMRI activity associated with Amyg-EFP modulation

To unveil the activation network associated with the Amyg-EFP modulation during emotional stimulation, we performed a linear regression of the BOLD signal to the simultaneously acquired Amyg-EFP time-course. This analysis aimed to test our primary hypothesis that the Amyg-EFP would correspond to a brain network involved in emotion processing.

First, we examined the right amygdala, which was used for the original Amyg-EFP modeling [25] and defined here by the AAL3 atlas [43]. As expected, voxels within the right amygdala exhibited above-chance coupling (*p* < 0.05, FDR-corrected) with the Amyg-EFP signal. This was consistently observed across all three naturalistic tasks (video clips, movie scenes and music) in both the test group (*n* = 36) and an independent replication sample (*n* = 35) (Fig. 2A).

Second, a whole-brain fMRI analysis revealed a network of regions significantly associated (*p* < 0.05, FDR-corrected) with Amyg-EFP signal fluctuations, across all three tasks (Fig. 2B). This revealed a distributed associated network further examined in the following analyses.

### Differential network activations associated with Amyg-EFP and VS-EFP modulation

To assess the neuroanatomical specificity of distributed fMRI activity (i.e. network) associated with Amyg-EFP modulation during emotional stimulation, we compared the regression effect for Amyg-EFP with the regression effect for the control EFP informed by fMRI activity in the ventral striatum (VS-EFP). We focused on the movie scene task for this analysis due to its rich, naturalistic content and longer duration, which provides the optimal sensitivity range to track activity fluctuations of various brain networks at once. This allowed for a direct comparison between the Amyg-EFP and the VS-EFP, which both responded dynamically to the movie stimuli.

**Fig. 3:**
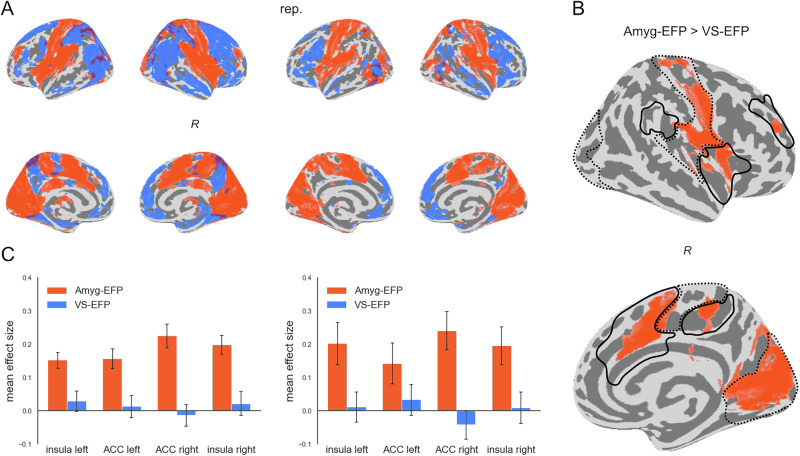
Whole-brain activations coupled with Amyg-EFP compared to VS-EFP signals. Results of linear regression of voxel-wise BOLD to EFP time-course for the Amyg-EFP and VS-EFP during passive viewing of movie scenes. (**A**) Above-chance voxels (*P* < 0.05, FDR corrected) associated with the Amyg-EFP (orange) and VS-EFP (blue), overlayed on an inflated brain template image. (**B**) Voxels showing significantly higher (*P* < 0.05, FDR corrected) GLM effect sizes for regression to the Amyg-EFP than to the VS-EFP were observed mainly in salience-network regions (black outline) and sensory (visual, auditory, somato-motor) regions (dotted outline), as defined by Schaefer et al., (2018) [65]. (**C**) Mean effect size in major nodes of the salience network [65], for regression to the Amyg-EFP (orange) and to the VS-EFP (blue), presented as mean ± SEM across subjects. Replication data presented in the second column, denoted ‘rep’.

Two distinct networks emerged when overlaying statistically significant voxels corresponding to each of the EFPs (*p* < 0.05, FDR corrected), from both participant groups (Fig. 3A). When directly contrasting the regression effects, voxels displaying significantly higher effect sizes for the Amyg-EFP relative to the VS-EFP were concentrated in salience network regions, especially the insula and dorsal anterior cingulate cortex (dACC), as well as in sensory cortices (Fig. 3B; Amyg-EFP > VS-EFP, *p* < 0.025, FDR corrected). Extraction of the mean effect sizes illustrates the stronger coupling between the Amyg-EFP and core salience network nodes compared to the VS-EFP (Amyg-EFP: mean β = 0.19, VS-EFP: mean β = 0.01)(Fig. 3C). Notably, a different activation map emerged when contrasting the VS-EFP over the Amyg-EFP (Supplementary Figure S1; VS-EFP > Amyg-EFP, *p* < 0.025, FDR corrected).

### Amyg-EFP correlations with amygdala subregions defined individually by salience coactivation

**Fig. 4:**
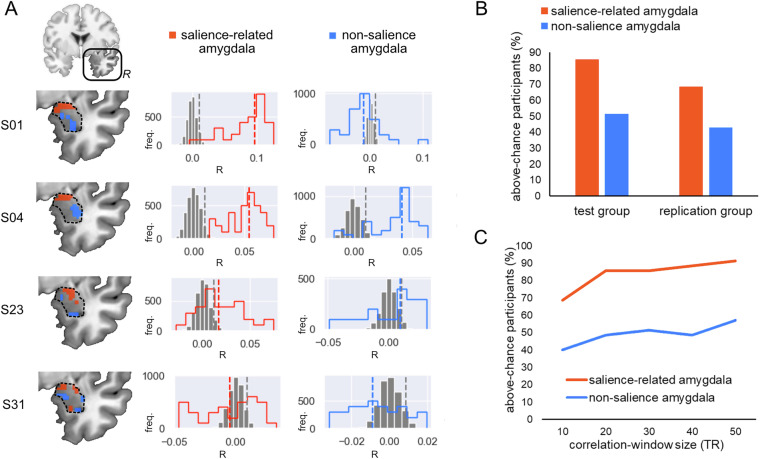
Nonparametric testing of the correlation distribution within functional amygdala clusters. Voxel-wise correlations between right-amygdala BOLD and Amyg-EFP signals (during passive movie viewing) were calculated along a sliding window of the run time-course and averaged across windows. To generate the null distribution the same procedure was repeated 100 times with shuffled data. (**A**) Results obtained from four example participants. Left panel presents coronal anatomical views of the right amygdala (dotted outline), highlighting functional salience-related (orange) and non-salience (blue) clusters, defined individually by high or low coactivation with a salience network seed in a separate task. The middle and right panels present the null correlation distribution (gray) and real correlation distribution (x100 for visualization) in the salience-related amygdala cluster (orange) and non-salience amygdala cluster (blue), per participant. Colored dashed lines denote the median of the real distribution, and gray dashed lines denote the 95th percentile of the null distribution (chance level). Above-chance participants were determined by a real-distribution median higher than the 95^th^ percentile of the null distribution. (**B**) Percentage of above-chance participants (as defined above) in each functional amygdala subregion, in the test group (left) and replication (right), for correlations tested along a 30-TR sliding window. (**C**) Percentage of above-chance participants (as defined above) in each of the functional amygdala subregions for sliding-window sizes ranging between 10 and 50 TR.

To further characterize the differential sensitivity of the Amyg-EFP to salience-related activity within the amygdala, we defined two functional amygdala subregions individually for each participant (see 4 different examples in Fig. 4A). Functional ROI selection within the amygdala was based on coactivation with a salience network seed during fear-tagged video clips (task condition selected to maximize salience signal to noise ratio). A “salience-related” amygdala cluster was defined by high seed coactivation (correlation above 85th percentile and R > 0.2) and a “non-salience” cluster was defined by low seed coactivation (correlation below 15th percentile and R < 0.2).

To examine each participant’s correspondence between Amyg-EFP and salience-related amygdala dynamics, we performed a nonparametric test of the correlation distribution within each of the predefined clusters. We used the movie scene data for this analysis due to its longer duration than the fear clips (used for ROI selection) and content variability, providing a dynamic response range required for reliable estimates of the correlations. The real distribution of sliding-window correlations between Amyg-EFP and BOLD time-courses was compared to a shuffled-windows null distribution. Results using a sliding window of 30-TR (45 sec) revealed a higher rate of above-chance correlations with the Amyg-EFP in the salience-related amygdala cluster than in the non-salience cluster (Fig. 4B). Specifically, in the salience-related cluster, 86% of participants (69% in the replication sample) exhibited above-chance correlations (median of real > 95th percentile of null) with the Amyg-EFP. By contrast, in the non-salience amygdala cluster, 51% of participants (43% in the replication sample) exhibited above-chance correlations with the Amyg-EFP. This pattern was consistently observed across different sliding-window sizes, ranging between 10 and 50 TR (Fig. 4C). These results demonstrate increased specificity of Amyg-EFP for salience-related subregions within the amygdala.

### Amyg-EFP correlation with individual emotional reactivity indices

To test the difference in individual reactivity to each of the two movie scenes (Snakes and Farewell) we examined the behavioral response to each movie scene in emotional intensity ratings collected outside the scanner. Henceforth, we will use the term emotional reactivity to refer to this measure. We used a reactivity index (see Methods) to capture variability in absolute range of emotional response rather than the valence of negative or positive value, based on previously demonstrated amygdala sensitivity to emotional reactivity (rather than valence) [45].

Results revealed a significant difference in emotional reactivity index between the two scenes across subjects (T = 2.28, *P* = 0.026), with higher reactivity to the Snakes scene than Farewell scene. This nicely mirrored the difference in Amyg-EFP activation amplitude between the two corresponding movie scenes (T = 3.44, *P* = 0.001), also showing higher response during the Snakes scene. Emotional reactivity was correlated with Amyg-EFP activation amplitude across subjects (R = 0.26, *P* = 0.04), showing that the difference in Amyg-EFP signal response when viewing the two movie scenes, was associated with the difference in subjective emotional reactivity to these same scenes (Supplementary Figure S2). By contrast, the VS-EFP did not show a difference in activation amplitude between the two movie scenes (T = 0.33, *P* = 0.744) nor was it correlated with the emotional reactivity difference (R = −0.11, *P* = 0.379).

## Discussion

The present study provides novel insights into the distributed neural correlates of the Amyg-EFP, during naturalistic emotional processing. We found that the Amyg-EFP signal modulation corresponds to a distributed network of fMRI activations beyond the amygdala (Fig. 2). The consistency of this pattern across three types of naturalistic emotional stimuli suggests a robust relationship between Amyg-EFP and this fMRI activation pattern. Particularly, the Amyg-EFP corresponded to sensory network activations and key nodes of the salience network (Fig. 3), known to be involved in emotional reactivity and modulation; two necessary subprocesses in emotion regulation [20]. Importantly, we show that salience network coupling was specific to the Amyg-EFP, whereas a control EFP derived from the ventral striatum was associated with a different activation topography. In addition, our results replicate previous studies validating the association of the Amyg-EFP with activations within the amygdala itself [22, 24, 25], and extend them by showing that subregions of the amygdala that are more functionally connected with salience network activity, are also more highly correlated with Amyg-EFP modulation during emotional stimulation (Fig. 4). Although in the current study we did not measure emotion regulation processes directly, the association of the Amyg-EFP with individual emotional ratings indicates its relevance for emotional reactivity, a necessary subprocess in emotion regulation [20]. Together, these findings suggest that Amyg-EFP modulation during naturalistic emotional stimulation corresponds to salience-network dynamics within the amygdala and beyond. Thereby, these findings extend our view of the neural correlates of Amyg-EFP, from its established amygdala correspondence to a broader network-level functionality.

The salience network, primarily comprised of the bilateral anterior insula and dACC (and adjacent pre-supplementary motor area), plays a crucial role in detecting salient stimuli and coordinating appropriate responses to these stimuli [44, 46]. The anterior insula in particular is thought to integrate interoceptive information associated with emotional salience to generate subjective feeling states [47]. The dACC is involved in conflict processing that may trigger top-down regulatory processes [48]. In the context of emotion regulation, the salience network as a whole facilitates the detection of emotionally salient information (i.e. reactivity) and may initiate regulatory processes by engaging other networks such as the central executive network (i.e. modulation) [49]. Though the current study did not directly measure emotion regulation abilities, the correspondence between Amyg-EFP amplitude and emotional reactivity ratings of the movie stimuli, indicates the relevance of Amyg-EFP signal modulation in the current data to emotional reactivity processing. Given the importance of the salience network in emotion regulation, the coupling of Amyg-EFP with salience-network related processing further highlights the relevance of Amyg-EFP for emotion reactivity.

Within the theoretical framework of emotion-regulation systems, the Amyg-EFP’s association with the salience network suggests that it may capture emotional reactivity and its corresponding neural circuits as a subprocess of emotion regulation [20, 50]. Specifically, the emotion regulation model proposed by Etkin et al. [20] stipulates that the amygdala, insula and dACC are all critically involved in emotional reactivity and participate in implicit processes such as salience detection and attention allocation. Supporting this idea, recent evidence shows that when viewing negative emotional images, the amygdala exerts an excitatory influence on salience network regions, while the periaqueductal gray provides inhibitory modulation on both the salience network and the amygdala [51]. This demonstrates the amygdala’s role in facilitating emotional reactivity to affective cues within the context of a complex regulation circuit. Here, the Amyg-EFP’s association with emotional ratings, the salience network and the amygdala, indicates its relevance to this implicit regulation mechanism. Notably, a similar circuit involving the amygdala and salience network regions was previously hypothesized to explain successful self-neuromodulation during neurofeedback [17], thus future studies using an active regulation task should examine the Amyg-EFP also in the context of explicit regulation mechanisms [19].

In addition to the main findings, our study revealed a segregation within the amygdala region, with distinct subregions showing differential coactivation with the salience network. This observation aligns with Gothard’s work [52] on multidimensional processing in the amygdala, which emphasizes the diverse functional roles of amygdala subregions. It suggested that neurons in the amygdala integrate social, sensory, and emotional information into overlapping multidimensional representations, from which salience arises as contextually-relevant features highlighted across inputs. The functional amygdala cluster we found to be connected to the salience network activity may be involved in processing various types of salient affective information, such as threat indicators, facial and vocal expressions, learned contextual cues, etc. This aligns with the amygdala’s known role in integrating multi-modal affective information and its involvement in salience processing [52], which is consistent with the use of naturalistic multi-modal stimulation in this study. This segregation highlights the complex and nuanced role of the amygdala in emotional processing and its interaction with large-scale brain networks that are known to be involved in emotional processing.

The clinical relevance of our findings is underscored by mounting research implicating dysfunction of the amygdala and salience network as a shared neural substrate underlying emotion dysregulation across a range of psychiatric disorders (i.e. trans-diagnostically) [53–58]. Importantly, alterations in amygdala and salience network activity have been linked to anxiety and stress-related disorders such as PTSD [56, 58]. In schizophrenia [52, 53], salience network regions exhibited hypo-activation during rest but hyper-activation in response to irrelevant stimuli [59, 60]. Interestingly, aberrant salience processing has also been proposed as a key mechanism in chronic somatic conditions characterized by emotion dysregulation, such as chronic pain in fibromyalgia [61]. Recent evidence suggests that similar mechanisms may be at play in other somatic conditions known as psychosomatic, like irritable bowel syndrome, where altered functional connectivity within the salience network has been observed [62]. While the specific clinical manifestations differ, these findings suggest potential common neural substrates, particularly the amygdala and salience network, which could be targeted therapeutically across mental and psychosomatic disorders by Amyg-EFP NF, as already demonstrated for some of these disorders [22, 27, 29]. Notably, given that some psychiatric conditions are characterized by blunted rather than heightened reactivity (e.g., anhedonia in depression; [63]), they may theoretically benefit from upregulation rather than downregulation training previously tested in disorders with hyperreactivity (e.g., PTSD; [64]).

By tracking amygdala dynamics embedded in the salience-emotion circuitry, the Amyg-EFP may act as a proxy for amygdala activity associated with emotional reactivity in psychopathology [9, 22, 32]. This has important implications for NF interventions, particularly in the context of process-guided NF, inspired by RDoC [1] as proposed by Lubianiker et al. [9]. By this idea, in order to address the underlying mechanism of a psychiatric disorder, NF should target a specific mental process by training to self-modulate its associated neural system. This approach is suggested to lead to more precise and personalized interventions [3]. Accordingly, Amyg-EFP NF can be conceptualized as a process-guided NF approach targeting emotional reactivity and its associated neural network, as has been proposed for amygdala-guided NF in general [17]. Indeed, recent work by Zopfs et al. [26] has explored potential relationships between the Amyg-EFP and various neural systems within the RDoC framework, under active fMRI-NF in a patient cohort that had partially been trained in Amyg-EFP NF. They found associations between Amyg-EFP and fMRI activation in the amygdala, dACC and sensory networks, as also seen here in healthy individuals when reacting to emotional stimuli. The current study extends our understanding of Amyg-EFP neural correlates by characterizing its associated network of brain activation, independent of activations induced by self-neuromodulation processes during NF [19, 53]. Ultimately, the Amyg-EFP could be relevant to both emotional modulation and emotional reactivity, based on its association with NF training during active modulation in previous work [22, 32], and with salience-network activity during naturalistic emotional stimulation found here.

We acknowledge that alternative approaches exist for developing neural measures of emotional processing. For example, decoded neurofeedback approaches use machine learning to extract task-specific patterns from brain activity, potentially offering more direct cognitive specificity [17]. Alternatively, behavioral marker calibration approaches validate neural measures against well-characterized behavioral tasks that isolate specific cognitive processes [19]. While these approaches offer advantages in terms of process specificity, our fMRI-informed approach provides unique insights into neuroanatomically defined correlates of an EEG signature that has already demonstrated therapeutic utility in multiple clinical populations. The current network-level understanding helps bridge the gap between scalable EEG measurement and the complex neural circuits underlying emotional processing. This was achieved by using naturalistic emotional stimulation along with the measurement of individual emotional reactivity, indicating the relevance of Amyg-EFP for this subprocess of emotion regulation. This is important for guiding future studies that will examine the neural changes associated with clinical effects of Amyg-EFP NF.

Finally, while providing valuable insights, a few limitations of the current study point towards potential future directions. First, we examined Amyg-EFP coupling during passive processing of naturalistic emotional stimuli, and the correlational nature of our findings precludes direct inferences about causal relationships between the Amyg-EFP modulation and engagement of emotion regulation processes. Future approaches employing active emotion regulation tasks, NF or prospective assessments before and after self-neuromodulation could help elucidate the directionality of the observed relationships, to provide more direct insights distinguishing between implicit and explicit regulation processes affecting the Amyg-EFP signal. Second, without preregistration, our focus on a specific task (particularly the movie scenes) for EFP comparisons may be viewed as an analytical limitation, though partially alleviated by the replication of these effects in the second group. Finally, the current work was performed on healthy participants, limiting generalizability. To enhance the clinical validity of our findings future studies should employ similar analyses on mixed data obtained from clinical and healthy populations.

In conclusion, this study presents the first comprehensive investigation of the distributed neural correlates of the Amyg-EFP model during naturalistic emotional stimulation. Particularly, our findings reveal that Amyg-EFP neural correlates extend beyond the amygdala, to the salience network at large, while at the same time highlighting the specificity of Amyg-EFP to salience-related activity within the amygdala itself. The correlation of the Amyg-EFP with individual emotional reactivity provides a cognitive context for understanding the functional relevance of these findings for emotional processing. Together, these findings tie amygdala activity captured by the Amyg-EFP during emotional processing, with broader network dynamics that might also be relevant for aspects of emotion regulation, particularly emotional reactivity. The demonstrated neural correlates of the Amyg-EFP, together with its clinical utility in NF, open new avenues for developing and refining scalable yet precise brain-based interventions for disorders of emotional dysregulation. Moreover, it highlights the potential of the EFP approach for translating neuroimaging insights into brain-based interventions, representing an important step towards realizing the promise of precision psychiatry.

## Supplementary information

Supplemental Material

Supplementary Figure S1: Neural network specificity of VS-EFP compared to Amyg-EFP

Supplementary Figure S2: Correlation between Amyg-EFP activation amplitude and subjective emotional reactivity

## Data Availability

The fMRI analysis code will be made available from the corresponding author upon reasonable request.

## Data availability

The human neuroimaging datasets generated and/or analyzed in this study are not publicly available as they are the intellectual property of GrayMatters Health. Data may be made available from GrayMatters Health upon reasonable request, subject to review under the company’s data-governance, privacy, and intellectual-property policies. Data requests should be addressed to Adar Shani at adar@graymatters-health.com.

## References

[1] Insel T, Cuthbert B, Garvey M, Heinssen R, Pine DS, Quinn K, et al. Research Domain Criteria (RDoC): toward a new classification framework for research on mental disorders. Am J Psychiatry. 2010;167:748–51. 20595427 10.1176/appi.ajp.2010.09091379

[2] Cuthbert BN, Insel TR. Toward the future of psychiatric diagnosis: the seven pillars of RDoC. BMC Med. 2013;11:126. 23672542 10.1186/1741-7015-11-126 PMC3653747

[3] Sanislow CA, Ferrante M, Pacheco J, Rudorfer MV, Morris SE. Advancing translational research using NIMH research domain criteria and computational methods. Neuron. 2019;101:779–82. 30844398 10.1016/j.neuron.2019.02.024

[4] Williams LM. Precision psychiatry: a neural circuit taxonomy for depression and anxiety. Lancet Psychiatry. 2016;3:472–80. 27150382 10.1016/S2215-0366(15)00579-9 PMC4922884

[5] Davis KD, Aghaeepour N, Ahn AH, Angst MS, Borsook D, Brenton A, et al. Discovery and validation of biomarkers to aid the development of safe and effective pain therapeutics: challenges and opportunities. Nat Rev Neurol. 2020;16:381–400. 32541893 10.1038/s41582-020-0362-2 PMC7326705

[6] Kraguljac NV, McDonald WM, Widge AS, Rodriguez CI, Tohen M, Nemeroff CB. Neuroimaging biomarkers in schizophrenia. Am J Psychiatry. 2021;178:appi.ajp.2020.2. 10.1176/appi.ajp.2020.20030340 PMC8222104 33397140

[7] Quinlan EB, Banaschewski T, Barker GJ, Bokde ALW, Bromberg U, Büchel C, et al. Identifying biological markers for improved precision medicine in psychiatry. Mol Psychiatry. 2019;25:243–53. 31676814 10.1038/s41380-019-0555-5 PMC6978138

[8] Woo C-W, Chang LJ, Lindquist MA, Wager TD. Building better biomarkers: brain models in translational neuroimaging. Nat Neurosci. 2017;20:365–77. 28230847 10.1038/nn.4478 PMC5988350

[9] Lubianiker N, Goldway N, Fruchtman-Steinbok T, Paret C, Keynan JN, Singer N, et al. Process-based framework for precise neuromodulation. Nat Hum Behav. 2019;3:436–45. 30988481 10.1038/s41562-019-0573-y

[10] Gross JJ. Emotion regulation: Current status and future prospects. Psychol Inq. 2015;26:1–26.

[11] Fernandez KC, Jazaieri H, Gross JJ. Emotion regulation: a transdiagnostic perspective on a new RDoC domain. Cognit Ther Res. 2016;40:426–40. 27524846 10.1007/s10608-016-9772-2 PMC4979607

[12] Kring AM, Sloan DM. Emotion Regulation and Psychopathology: A Transdiagnostic Approach to Etiology and Treatment. Guilford Press; 2009.

[13] Cisler JM, Sigel BA, Kramer TL, Smitherman S, Vanderzee K, Pemberton J, et al. Amygdala response predicts trajectory of symptom reduction during Trauma-Focused Cognitive-Behavioral Therapy among adolescent girls with PTSD. J Psychiatr Res. 2015;71:33–40. 26522869 10.1016/j.jpsychires.2015.09.011 PMC4826076

[14] Goldin PR, Ziv M, Jazaieri H, Hahn K, Heimberg R, Gross JJ. Impact of cognitive behavioral therapy for social anxiety disorder on the neural dynamics of cognitive reappraisal of negative self-beliefs. JAMA Psychiatry. 2013;70:1048. 23945981 10.1001/jamapsychiatry.2013.234 PMC4141477

[15] Zilverstand A, Parvaz MA, Goldstein RZ. Neuroimaging cognitive reappraisal in clinical populations to define neural targets for enhancing emotion regulation. A systematic review. Neuroimage. 2017;151:105–16. 27288319 10.1016/j.neuroimage.2016.06.009 PMC5145785

[16] Thibault RT, MacPherson A, Lifshitz M, Roth RR, Raz A. Neurofeedback with fMRI: A critical systematic review. Neuroimage. 2018;172:786–807. 29288868 10.1016/j.neuroimage.2017.12.071

[17] Paret C, Hendler T. Live from the ‘regulating brain’: Harnessing the brain to change emotion. Emotion. 2020;20:126–31. 31961191 10.1037/emo0000674

[18] Goldway N, Strauss I, Keynan JN, Hellrung L, Horstmann A, Paret C, et al. Feasibility and utility of amygdala neurofeedback. Neurosci Biobehav Rev. 2022;138:104694–104694. 35623447 10.1016/j.neubiorev.2022.104694

[19] Gross J. Handbook of emotion regulation. 2nd ed. New York: The Guilford Press; 2014.

[20] Etkin A, Büchel C, Gross JJ. The neural bases of emotion regulation. Nat Rev Neurosci. 2015;16:693–700. 26481098 10.1038/nrn4044

[21] Ferrante M, Gordon JA. Computational phenotyping and longitudinal dynamics to inform clinical decision-making in psychiatry. Neuropsychopharmacology. 2020;46:243–4. 10.1038/s41386-020-00852-z PMC7688969 32908241

[22] Keynan JN, Cohen A, Jackont G, Green N, Goldway N, Davidov A, et al. Electrical fingerprint of the amygdala guides neurofeedback training for stress resilience. Nat Hum Behav. 2018;3:63–73. 30932053 10.1038/s41562-018-0484-3

[23] Meir-Hasson Y, Keynan JN, Kinreich S, Jackont G, Cohen A, Podlipsky-Klovatch I, et al. One-class FMRI-Inspired EEG model for self-regulation training. PLoS ONE. 2016;11:e0154968–e0154968. 27163677 10.1371/journal.pone.0154968 PMC4862623

[24] Keynan JN, Meir-Hasson Y, Gilam G, Cohen A, Jackont G, Kinreich S, et al. Limbic activity modulation guided by functional magnetic resonance imaging–inspired electroencephalography improves implicit emotion regulation. Biol Psychiatry. 2016;80:490–6. 26996601 10.1016/j.biopsych.2015.12.024

[25] Meir-Hasson Y, Kinreich S, Podlipsky I, Hendler T, Intrator N. An EEG Finger-Print of fMRI deep regional activation. Neuroimage. 2014;102:128–41. 24246494 10.1016/j.neuroimage.2013.11.004

[26] Zopfs M, Jindrová M, Gurevitch G, Keynan JN, Hendler T, Baumeister S, et al. Amygdala-related electrical fingerprint is modulated with neurofeedback training and correlates with deep-brain activation: proof-of-concept in borderline personality disorder. Psychol Med. 2023;54:1651–60. 38131344 10.1017/S0033291723003549

[27] Fruchter E, Goldenthal N, Adler LA, Gross R, Harel EV, Deutsch L, et al. Amygdala-derived-EEG-fMRI-Pattern Neurofeedback for the treatment of chronic post-traumatic stress disorder. A prospective, multicenter, multinational study evaluating clinical efficacy. Psychiatry Res. 2024;333:115711–115711. 38325159 10.1016/j.psychres.2023.115711

[28] Fruchtman-Steinbok T, Keynan JN, Cohen A, Jaljuli I, Mermelstein S, Drori G, et al. Amygdala electrical-finger-print (AmygEFP) NeuroFeedback guided by individually-tailored Trauma script for post-traumatic stress disorder: Proof-of-concept. NeuroImage: Clinical. 2021;32:102859. 34689055 10.1016/j.nicl.2021.102859 PMC8551212

[29] Goldway N, Ablin JN, Lubin O, Zamir Y, Keynan JN, Or-Borichev A, et al. Volitional limbic neuromodulation exerts a beneficial clinical effect on Fibromyalgia. Neuroimage. 2019;186:758–70. 30408596 10.1016/j.neuroimage.2018.11.001

[30] Tene O, Bleich Cohen M, Helpman L, Fine NB, Halevy A, Goldway N, et al. Limbic self‐neuromodulation as a novel treatment option for emotional dysregulation in premenstrual dysphoric disorder (PMDD); a proof‐of‐concept study. Psychiatry Clin Neurosci. 2023;77:550–8. 37354437 10.1111/pcn.13574

[31] Adler LA, Anbarasan D, Leon T, Sardoff T, Descorbeth O, Cho D, et al. Pilot study of prism EFP NeuroFeedback in adult ADHD. J Atten Disord. 2023;28:905–12. 38152997 10.1177/10870547231215283

[32] Gurevitch G, Lubianiker N, Markovits T, Or-Borichev A, Sharon H, Fine NB, et al. Amygdala self-neuromodulation capacity as a window for process-related network recruitment. Philos Trans R Soc Lond B Biol Sci. 2024;379:20240186. 39428877 10.1098/rstb.2024.0186 PMC11491848

[33] Cowen AS, Keltner D. Self-report captures 27 distinct categories of emotion bridged by continuous gradients. Proc Natl Acad Sci. 2017;114:E7900–E7909. 28874542 10.1073/pnas.1702247114 PMC5617253

[34] Raz G, Winetraub Y, Jacob Y, Kinreich S, Maron-Katz A, Shaham G, et al. Portraying emotions at their unfolding: a multilayered approach for probing dynamics of neural networks. Neuroimage. 2012;60:1448–61. 22285693 10.1016/j.neuroimage.2011.12.084

[35] Singer N, Jacoby N, Lin T, Raz G, Shpigelman L, Gilam G, et al. Common modulation of limbic network activation underlies musical emotions as they unfold. Neuroimage. 2016;141:517–29. 27389788 10.1016/j.neuroimage.2016.07.002

[36] Xu J, Moeller S, Auerbach EJ, Strupp J, Smith SM, Feinberg DA, et al. Evaluation of slice accelerations using multiband echo planar imaging at 3T. Neuroimage. 2013;83:991–1001. 23899722 10.1016/j.neuroimage.2013.07.055 PMC3815955

[37] Andersson JLR, Skare S, Ashburner J. How to correct susceptibility distortions in spin-echo echo-planar images: application to diffusion tensor imaging. Neuroimage. 2003;20:870–88. 14568458 10.1016/S1053-8119(03)00336-7

[38] Esteban O, Markiewicz CJ, Blair RW, Moodie CA, Isik AI, Erramuzpe A, et al. fMRIPrep: a robust preprocessing pipeline for functional MRI. Nat Methods. 2019;16:111–6. 30532080 10.1038/s41592-018-0235-4 PMC6319393

[39] Allen PJ, Josephs O, Turner R. a method for removing imaging artifact from continuous EEG recorded during functional MRI. Neuroimage. 2000;12:230–9. 10913328 10.1006/nimg.2000.0599

[40] Allen PA, Polizzi G, Krakow K, Fish DR, Lemieux L. Identification of EEG events in the MR scanner: the problem of pulse artifact and a method for its subtraction. Neuroimage. 1998;8:229–39. 9758737 10.1006/nimg.1998.0361

[41] Singer N, Poker G, Dunsky N, Nemni S, Reznik Balter S, Doron M, et al. Development and validation of an fMRI-informed EEG model of reward-related ventral striatum activation. Neuroimage. 2023;276:120183–120183. 37225112 10.1016/j.neuroimage.2023.120183 PMC10300238

[42] Abraham A, Pedregosa F, Eickenberg M, Gervais P, Mueller A, Kossaifi J, et al. Machine learning for neuroimaging with scikit-learn. Front Neuroinform. 2014;8:14. 24600388 10.3389/fninf.2014.00014 PMC3930868

[43] Rolls ET, Huang C-C, Lin C-P, Feng J, Joliot M. Automated anatomical labelling atlas 3. Neuroimage. 2020;206:116189. 31521825 10.1016/j.neuroimage.2019.116189

[44] Menon VP. Large-Scale Functional Brain Organization. Brain Mapping: An Encyclopedic Reference. 2. Elsevier BV; 2015. p. 449–59.

[45] Kahn I, Yeshurun Y, Rotshtein P, Fried I, Ben-Bashat D, Hendler T. The role of the amygdala in signaling prospective outcome of choice. Neuron. 2002;33:983–94. 11906703 10.1016/s0896-6273(02)00626-8

[46] Seeley WW. The salience network: a neural system for perceiving and responding to homeostatic demands. J Neurosci. 2019;39:9878–82. 31676604 10.1523/JNEUROSCI.1138-17.2019 PMC6978945

[47] Craig AD. How do you feel — now? The anterior insula and human awareness. Nat Rev Neurosci. 2009;10:59–70. 19096369 10.1038/nrn2555

[48] Botvinick MM, Cohen JD, Carter CS. Conflict monitoring and anterior cingulate cortex: an update. Trends Cogn Sci. 2004;8:539–46. 15556023 10.1016/j.tics.2004.10.003

[49] Ochsner KN, Silvers JA, Buhle JT. Functional imaging studies of emotion regulation: a synthetic review and evolving model of the cognitive control of emotion. Ann N Y Acad Sci. 2012;1251:E1–E24. 23025352 10.1111/j.1749-6632.2012.06751.x PMC4133790

[50] Braunstein LM, Gross JJ, Ochsner KN. Explicit and implicit emotion regulation: a multi-level framework. Soc Cogn Affect Neurosci. 2017;12:1545–57. 28981910 10.1093/scan/nsx096 PMC5647798

[51] Ince S, Steward T, Harrison BJ, Jamieson AJ, Davey CG, Agathos JA, et al. Subcortical contributions to salience network functioning during negative emotional processing. Neuroimage. 2023;270:119964–119964. 36822252 10.1016/j.neuroimage.2023.119964

[52] Gothard KM. Multidimensional processing in the amygdala. Nat Rev Neurosci. 2020;21:565–75. 32839565 10.1038/s41583-020-0350-y PMC7714370

[53] Downar J, Blumberger DM, Daskalakis ZJ. The neural crossroads of psychiatric illness: an emerging target for brain stimulation. Trends Cogn Sci. 2016;20:107–20. 26655436 10.1016/j.tics.2015.10.007

[54] Goodkind M, Eickhoff SB, Oathes DJ, Jiang Y, Chang A, Jones-Hagata LB, et al. Identification of a common neurobiological substrate for mental illness. JAMA Psychiatry. 2015;72:305. 25651064 10.1001/jamapsychiatry.2014.2206 PMC4791058

[55] Schimmelpfennig J, Topczewski J, Zajkowski W, Jankowiak-Siuda K. The role of the salience network in cognitive and affective deficits. Front Hum Neurosci. 2023;17:1133367. 37020493 10.3389/fnhum.2023.1133367 PMC10067884

[56] Etkin A, Wager TD. Functional neuroimaging of anxiety: a meta-analysis of emotional processing in PTSD, social anxiety disorder, and specific phobia. Am J Psychiatry. 2007;164:1476–88. 17898336 10.1176/appi.ajp.2007.07030504 PMC3318959

[57] Hamilton JP, Etkin A, Furman DJ, Lemus MG, Johnson RF, Gotlib IH. Functional neuroimaging of major depressive disorder: a meta-analysis and new integration of baseline activation and neural response data. Am J Psychiatry. 2012;169:693–703. 22535198 10.1176/appi.ajp.2012.11071105 PMC11889638

[58] Sripada R, King A, Garfinkel S, Wang X, Sripada C, Welsh R, et al. Altered resting-state amygdala functional connectivity in men with posttraumatic stress disorder. J Psychiatry Neurosci. 2012;37:241–9. 22313617 10.1503/jpn.110069 PMC3380095

[59] Palaniyappan L, Simmonite M, White Thomas P, Liddle Elizabeth B, Liddle Peter F. Neural primacy of the salience processing system in schizophrenia. Neuron. 2013;79:814–28. 23972602 10.1016/j.neuron.2013.06.027 PMC3752973

[60] Menon V, Palaniyappan L, Supekar K. Integrative brain network and salience models of psychopathology and cognitive dysfunction in schizophrenia. Biol Psychiatry. 2022;94:108–20. 36702660 10.1016/j.biopsych.2022.09.029

[61] Pinto AM, Geenen R, Wager TD, Lumley MA, Häuser W, Kosek E, et al. Emotion regulation and the salience network: a hypothetical integrative model of fibromyalgia. Nat Rev Rheumatol. 2023;19:44–60. 36471023 10.1038/s41584-022-00873-6

[62] Qi R, Liu C, Ke J, Xu Q, Zhong J, Wang F, et al. Intrinsic brain abnormalities in irritable bowel syndrome and effect of anxiety and depression. Brain Imaging Behav. 2015;10:1127–34. 10.1007/s11682-015-9478-1 26556814

[63] Pizzagalli DA. Depression, stress, and anhedonia: toward a synthesis and integrated model. Annu Rev Clin Psychol. 2014;10:393–423. 24471371 10.1146/annurev-clinpsy-050212-185606 PMC3972338

[64] Nicholson AA, Rabellino D, Densmore M, Frewen PA, Paret C, Kluetsch R, et al. The neurobiology of emotion regulation in posttraumatic stress disorder: Amygdala downregulation via real‐time fMRI neurofeedback. Hum Brain Mapp. 2016;38:541–60. 27647695 10.1002/hbm.23402 PMC6866912

[65] Schaefer A, Kong R, Gordon EM, Laumann TO, Zuo X-N, Holmes AJ, et al. Local-global parcellation of the human cerebral cortex from intrinsic functional connectivity MRI. Cereb Cortex. 2017;28:3095–114. 10.1093/cercor/bhx179 PMC6095216 28981612

