# Supplementary material for: An fMRI-informed EEG model of the amygdala is associated with salience network dynamics during naturalistic emotional stimulation
Source: Mol Psychiatry. 2025 Dec 15;31(5):2520–9. doi: 10.1038/s41380-025-03418-x (PMC13099433; doi:10.1038/s41380-025-03418-x)
Supplement: Supplementary file 1 — Supplemental Material [file 41380_2025_3418_MOESM1_ESM.docx]

**Supplementary Materials – *An fMRI-informed EEG model of the amygdala is associated with salience network dynamics during naturalistic emotional stimulation***

**Supplementary Figure Legends**

**Supplementary Figure S1: Neural network specificity of VS-EFP compared to Amyg-EFP**

Results of linear regression showing voxels with significantly higher (P < 0.025, FDR corrected) GLM effect sizes for VS-EFP than Amyg-EFP during movie viewing. This corresponds with Figure 3B that presented the specificity of Amyg-EFP over VS-EFP. Together, the distinct network patterns confirm the neuroanatomical specificity of each EFP model.

**Supplementary Figure S2: Correlation between Amyg-EFP activation amplitude and subjective emotional reactivity**

Scatter-plot of the magnitudes of difference in response to the two movie scenes for each participant (n=61) in their Amyg-EFP activation (X axis) and in their emotional reactivity rating (Y axis). Results show a positive correlation (R = 0.26, P = 0.04) between Amyg-EFP activation and emotional reactivity, demonstrating the relevance of Amyg-EFP to emotional processing.

**Supplementary Methods**

Preprocessing of fMRI data using fMRIPrep 22.0.0:

First, a reference volume and its skull-stripped version were generated using a custom methodology of fMRIPrep. Head-motion parameters with respect to the BOLD reference (transformation matrices, and six corresponding rotation and translation parameters) are estimated before any spatiotemporal filtering using `mcflirt` [FSL 6.0.5.1:57b01774, @mcflirt]. BOLD runs were slice-time corrected to 0.955s (0.5 of slice acquisition range 0s-1.91s) using `3dTshift` from AFNI [@afni, RRID:SCR_005927]. The BOLD time-series (including slice-timing correction when applied) were resampled onto their original, native space by applying the transforms to correct for head-motion. These resampled BOLD time-series will be referred to as preprocessed BOLD in original space, or just preprocessed BOLD. The BOLD reference was then co-registered to the T1w reference using `mri_coreg` (FreeSurfer) followed by `flirt` [FSL 6.0.5.1:57b01774, @flirt] with the boundary-based registration [@bbr] cost-function. Co-registration was configured with six degrees of freedom. Several confounding time-series were calculated based on the preprocessed BOLD: framewise displacement (FD), DVARS and three region-wise global signals. FD was computed using two formulations following Power (absolute sum of relative motions, @power_fd_dvars) and Jenkinson (relative root mean square displacement between affines, @mcflirt). FD and DVARS are calculated for each functional run, both using their implementations in Nipype [following the definitions by @power_fd_dvars]. The three global signals are extracted within the CSF, the WM, and the whole-brain masks. Additionally, a set of physiological regressors were extracted to allow for component-based noise correction [CompCor, @compcor]. Principal components are estimated after high-pass filtering the preprocessed BOLD time-series (using a discrete cosine filter with 128s cut-off) for the two CompCor variants: temporal (tCompCor) and anatomical (aCompCor). tCompCor components are then calculated from the top 2% variable voxels within the brain mask. For aCompCor, three probabilistic masks (CSF, WM and combined CSF+WM) are generated in anatomical space. The implementation differs from that of Behzadi et al. in that instead of eroding the masks by 2 pixels on BOLD space, a mask of pixels that likely contain a volume fraction of GM is subtracted from the aCompCor masks. This mask is obtained by thresholding the corresponding partial volume map at 0.05, and it ensures components are not extracted from voxels containing a minimal fraction of GM. Finally, these masks are resampled into BOLD space and binarized by thresholding at 0.99 (as in the original implementation). Components are also calculated separately within the WM and CSF masks. For each CompCor decomposition, the k components with the largest singular values are retained, such that the retained components' time series are sufficient to explain 50 percent of variance across the nuisance mask (CSF, WM, combined, or temporal). The remaining components are dropped from consideration. The head-motion estimates calculated in the correction step were also placed within the corresponding confounds file. The confound time series derived from head motion estimates and global signals were expanded with the inclusion of temporal derivatives and quadratic terms for each [@confounds_satterthwaite_2013]. Frames that exceeded a threshold of 0.5 mm FD or 1.5 standardized DVARS were annotated as motion outliers. Additional nuisance timeseries are calculated by means of principal components analysis of the signal found within a thin band (crown) of voxels around the edge of the brain, as proposed by [@patriat_improved_2017]. The BOLD time-series were resampled into standard space, generating a preprocessed BOLD run in MNI152NLin2009cAsym space. First, a reference volume and its skull-stripped version were generated using a custom methodology of fMRIPrep. Automatic removal of motion artifacts using independent component analysis [ICA-AROMA, @aroma] was performed on the preprocessed BOLD on MNI space time-series after removal of non-steady state volumes and spatial smoothing with an isotropic, Gaussian kernel of 6mm FWHM (full-width half-maximum). Corresponding "non-aggresively" denoised runs were produced after such smoothing. Additionally, the "aggressive" noise-regressors were collected and placed in the corresponding confounds file. All resamplings can be performed with a single interpolation step by composing all the pertinent transformations (i.e. head-motion transform matrices, susceptibility distortion correction when available, and co-registrations to anatomical and output spaces). Gridded (volumetric) resamplings were performed using `antsApplyTransforms` (ANTs), configured with Lanczos interpolation to minimize the smoothing effects of other kernels [@lanczos]. Non-gridded (surface) resamplings were performed using `mri_vol2surf`(FreeSurfer).
