# Supplementary figures and images for: An fMRI-informed EEG model of the amygdala is associated with salience network dynamics during naturalistic emotional stimulation
Source: Mol Psychiatry. 2025 Dec 15;31(5):2520–9. doi: 10.1038/s41380-025-03418-x (PMC13099433; doi:10.1038/s41380-025-03418-x)

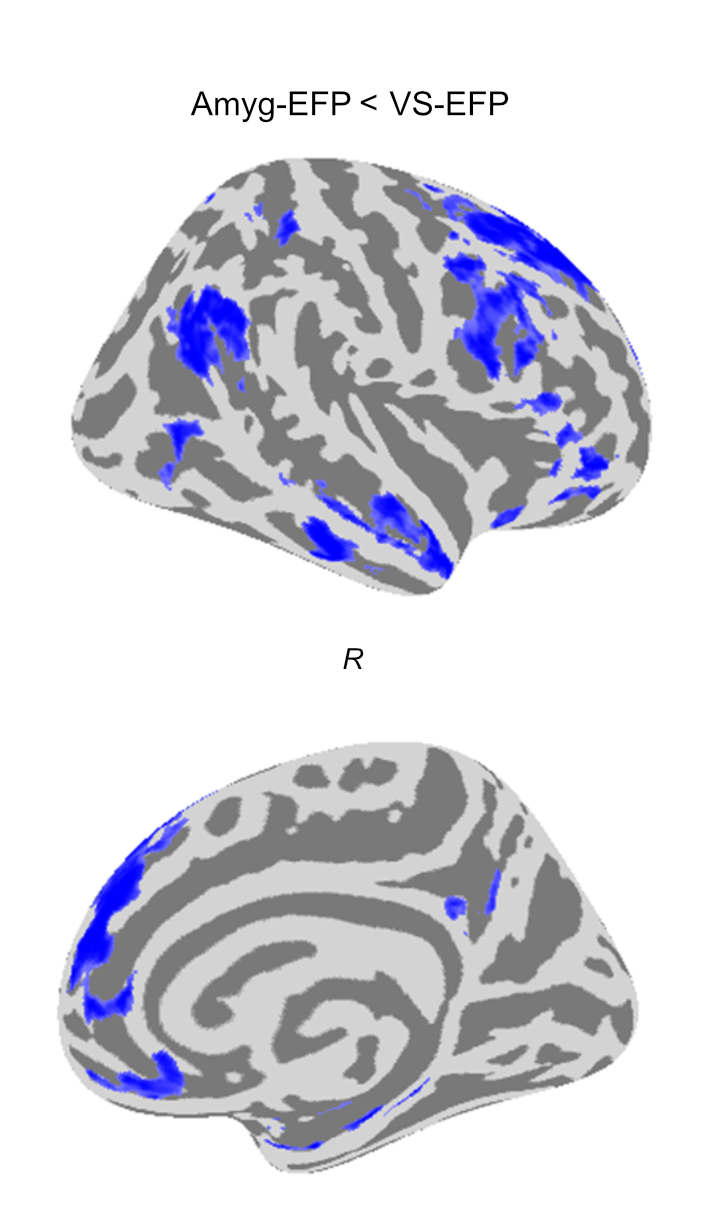

Supplement: Supplementary file 2 — Supplementary Figure S1: Neural network specificity of VS-EFP compared to Amyg-EFP [file 41380_2025_3418_MOESM2_ESM.png]

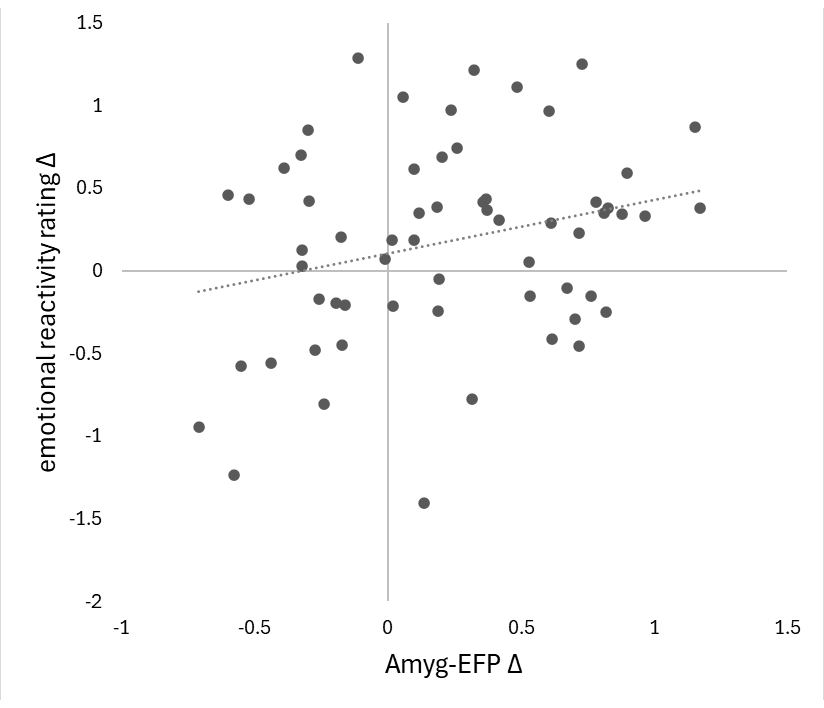

Supplement: Supplementary file 3 — Supplementary Figure S2: Correlation between Amyg-EFP activation amplitude and subjective emotional reactivity [file 41380_2025_3418_MOESM3_ESM.png]
